# Factors Associated With Anemia in Xavante Indigenous Children From Central Brazil

**DOI:** 10.1002/ajhb.70049

**Published:** 2025-05-06

**Authors:** Larissa de Lima Alves Paresque, Juliana de Bem Lignani, Rui Arantes, James R. Welch, Carlos E. A. Coimbra, Aline Alves Ferreira

**Affiliations:** ^1^ Instituto de Nutrição Josué de Castro Universidade Federal do Rio de Janeiro (UFRJ) Rio de Janeiro Brazil; ^2^ Instituto de Nutrição Universidade do Estado do Rio de Janeiro Rio de Janeiro Brazil; ^3^ Escola Nacional de Saúde Sérgio Arouca Fundação Oswaldo Cruz Rio de Janeiro Brazil

**Keywords:** anemia, child nutrition, indigenous health, social inequity, south American indigenous

## Abstract

**Objective:**

The objective was to evaluate the factors associated with anemia in Xavante children from the Pimentel Barbosa Indigenous Territory (IT) in Central Brazil through path analysis.

**Methods:**

A survey was conducted with children between 6 months and 5 years in the Pimentel Barbosa Indigenous Reserve in 2011. Hemoglobin levels, anthropometric measurements, and socioeconomic/demographic data were collected, and cut‐off points were recommended by the World Health Organization in 2024. A theoretical model was adapted from previous literature, and direct and indirect associations were evaluated on a theoretical‐scientific basis through path analysis. A significance level of 5% was considered.

**Results:**

Approximately 61.1% of the Indigenous children evaluated had anemia (33.7% < 5 years old had moderate/severe anemia and 78.1% < 2 years old had anemia). The final model presented an acceptable fit. Significant and direct relationships were observed between children's age (*β* = 0.460), the number of residents in the household (*β* = −0.143), the village group (*β* = −0.346) and hemoglobin levels.

**Conclusion:**

According to their hemoglobin levels, anemia was more prevalent in children under 2 years of age, as well as in children living in the most populous households and the newest village groups, than in the other groups. These findings point to the existence of socioeconomic, demographic, historical, and biological determinants of the prevalence of anemia. In addition, this study showed that Indigenous peoples in Brazil experience health inequities.

## 1 Introduction

According to the World Health Organization (WHO 2023), one quarter of the global population is estimated to be affected by anemia, particularly children under 5 years of age and women of childbearing age. In 2023, this condition affected 571 million women and 269 million children worldwide. In addition to its association with high rates of infant morbidity and mortality, anemia impairs cognitive and motor development, leads to low productivity, and causes fatigue (Stringhini et al. 2024). Half of the existing cases worldwide are caused by iron deficiency, with higher incidences in low‐ and middle‐income countries (Larson et al. 2024; Petry et al. 2016; WHO 2023), and iron deficiency is highly prevalent among certain ethnic groups, particularly in Latin American and African individuals. Among South American Indigenous peoples, the prevalence of anemia ranges from 19% to 100% in women and from 39% to 98% in children (De Louize et al. 2022).

The prevalence of anemia in Brazilian children has decreased over the past few years. According to the National Study on Food and Nutrition (ENANI), the most recent population‐based survey conducted with children under 5 years old in 2019, 10% of the included children had anemia (UFRJ 2021). This percentage is significantly lower than that reported in a previous population survey in 2006, in which 20.9% of children had anemia, despite the different blood collection methodologies used (Brasil 2009). However, these percentages still represent prevalences that are equivalent to those in countries with high levels of social inequality (Balarajan et al. 2011; Larson et al. 2024).

This situation is even worse for Indigenous children, and there are no available comparative data over the years. The only national survey that provides an overview of anemia in Indigenous children in Brazil was conducted in 2008/2009, and 51.2% of this population group had anemia (Leite et al. 2013). Despite being under‐investigated among the estimated 1 693 535 Indigenous people in Brazil (IBGE 2023), studies commonly indicate a prevalence of approximately 80% among children under 2 years of age in certain ethnic groups (Bresan et al. 2024; Ferreira et al. 2024, 2017; Rosas‐Jiménez et al. 2022). The Xavante people are considered in this high percentage. The latest publications on the prevalence of anemia in this ethnic group indicate that 50.8% of children have anemia, and among these children, 77.8% are under 2 years of age (Ferreira et al. 2017).

The understanding and analysis of factors associated with anemia in Indigenous children is another challenge in the fields of health and human biology. This assessment is further complicated by the distinct sociocultural, historical, and environmental contexts of this population, which can directly affect the nutritional status of children (Huda et al. 2018; Rosas‐Jiménez et al. 2022). The few studies conducted with Indigenous peoples in Brazil and South America that have evaluated the determinants of anemia highlighted that unfavorable social and environmental conditions are associated with nutritional issues (Ferreira et al. 2023; Leite et al. 2013; Rosas‐Jiménez et al. 2022). High rates of anemia have been linked to poor health conditions, a lack of basic sanitation, limited access to education, inadequate nutrition, and an unfavorable nutritional status (Ferreira et al. 2024; Larson et al. 2024). In addition to these factors, demographic, biological, maternal, and socioeconomic conditions are also described in the literature as being associated with childhood anemia among Indigenous populations (Ferreira et al. 2017; Larson et al. 2024; Leite et al. 2013).

Previous studies have typically focused on specific villages or population groups without including an entire Indigenous territory (IT) or ethnic group (Ferreira et al. 2017, 2024). Furthermore, the literature on Indigenous people's nutritional status, including anemia and its determinants, is often limited to descriptions or associations among only a few factors at a time (Ghosh 2023; Lício et al. 2016). The possibility of evaluating living conditions, considering their correlations with and mediating effects on sociodemographic variables and the historical processes of the group under study, may provide new insights into the complex determinants of anemia among Indigenous peoples. Therefore, this study aimed to evaluate the determinants of anemia in Xavante children from the Pimentel Barbosa IT in Central Brazil through path analysis, a technique that allowed for the visualization of relationships between variables and the identification of causal relationships of variables with anemia in a novel way.

## 2 Methods

### 2.1 Study Design and Setting

This was a cross‐sectional epidemiological study that used data from a population‐based health and nutrition survey conducted with the Xavante people of the Pimentel Barbosa reserve. The survey was carried out between July and August 2011, aiming to cover the entire population of the reserve, an unprecedented feature among studies with Indigenous peoples in Brazil. In the present study, information was collected from children aged between 6 months and 5 years. No specific sampling technique was used; all eligible children of both sexes were included in the study.

The Xavante Pimentel Barbosa reserve is located in the eastern part of the State of Mato Grosso in Central Brazil. This region is characterized by the Cerrado biome within the Legal Amazon, with distinct environmental features and sociocultural diversity. The Xavante are among the most populous Indigenous people, with approximately 25 600 individuals distributed across 10 ITs. The Xavante people belong to the Akuen linguistic group of the Macro‐Gê trunk; their self‐designation is A'uwe (“people”) and they constitute one of the largest Gê‐speaking ethnic groups in Central Brazil. Pimentel Barbosa Indigenous Reserve is the largest Xavante reserve, demarcated and recognized by the Federal Government since 1976. Permanent contact with national society began in the late 1940s, and the historical process of interaction with non‐Indigenous people, the resulting socioeconomic transformations, and their consequences for the health of the Xavante are well‐documented.

Like other Gê‐speaking Indigenous groups in Brazil, the Xavante were considered seminomadic by early anthropological scholars because they depended more on gathering and hunting than on agriculture for most of the year (Welch et al. 2013). In the 1970s and 1980s, the National Indian Foundation (FUNAI) inaugurated a mechanized rice cultivation project on Xavante lands, including Pimentel Barbosa, with the aim of selling the surplus produced on the regional market. But the Xavante project failed in a short space of time.

Many transformations have occurred and, since the 1990s, the Xavante have dedicated less time to agriculture and more time to obtaining wild resources within their reserve, especially hunting, fishing, and gathering fruits and tubers (Welch and Coimbra Jr. 2022). Conversely, the proximity of some Xavante communities to regional markets also facilitates access to and consumption of ultra‐processed foods. This phenomenon has been occurring continuously and intensively in recent years and has been observed not only among the Xavante but also in several other Indigenous groups in Brazil, influencing their dietary patterns.

Currently, the reserve has a health post and a local school that directly serves the communities of Pimentel Barbosa and Etênhiritipá. However, residents of other villages within the reserve must travel to nearby cities to access essential services such as healthcare and education. These cities have limited infrastructure and are underdeveloped, which may hinder adequate service provision to the Xavante Indigenous population. The education system contributes to community economies where schools are present, as is the case in the communities of Pimentel Barbosa and Etênhiritipá. Elementary schools follow the standards of Brazilian rural schools (Welch and Coimbra Jr. 2022).

At the time of the survey, the Pimentel Barbosa IT comprised 10 villages, with an estimated total population of 1466 Xavante individuals (19.2% were children under 5 years old) (Welch et al. 2013). Of the 10 villages that existed in the Pimentel Barbosa at the time, two did not participate due to their small populations, and one did not include individuals from the target population for this study.

### 2.2 Data

Socioeconomic, demographic, health, and nutritional data were collected from the Xavante people. Semistructured questionnaires, which are based on the First Indigenous Peoples Health and Nutrition Survey (Arantes et al. 2018), were used. These questionnaires were administered in the participants' homes by trained professionals, with the assistance of a Xavante translator when necessary.

Anthropometric data for the children and their biological mothers were collected by trained and standardized professionals, following the protocol of Lohman et al. (1988). For both the children and their mothers, weight was measured via a SECA 872 digital scale (Hamburg, Germany), with an accuracy of 0.1 kg and a maximum capacity of 150 kg. This device features a “mother/child” function, which allows the child's weight to be determined while being held, as well as the weight of the mother or guardian holding the child. The standing height and length of children under 2 years of age were measured with two SECA (SECA 416 and SECA 216, Hamburg, Germany) anthropometers specifically designed for measuring recumbent length and standing height, with a specificity of 0.1 cm (Lohman et al. 1988).

The weight‐for‐age *z* score (WAZ) was calculated for the children, and the body mass index (BMI) was calculated for the adults. The BMI cutoff points recommended by the WHO (2006) were used to classify the nutritional status of the mothers. For the WAZ assessment in children, Anthro software, provided by the WHO (2011), was used.

For blood collection, disposable lancets and an Accu‐Chek Softclix lancet device were used to obtain a drop of blood from the fingertips of the children and their biological mothers. The hemoglobin levels were measured via a Hemocue Hb 201+ device (Angelholm, Sweden). The classification of anemia in children and their mothers according to hemoglobin level followed the criteria proposed by the WHO (2024) (children 6–23 months, < 105 g/L hemoglobin concentration; children 24–59 months, < 110 g/L hemoglobin concentration).

### 2.3 Study Variables

Among the variables with data available in the survey database, age, sex, hospitalization frequency, and the height‐for‐age *z* score (HAZ) were selected for the children. Additionally, maternal variables such as age, BMI, anemia status, and serum hemoglobin level were selected. For both groups, per capita income, household population size, and village group affiliation were selected as variables. The selection of these variables was based on the researchers' previous experiences and prior publications related to the nutritional status of the Xavante (Arantes et al. 2018; Ferreira et al. 2017).

Based on the assessment of anthropometric, clinical, maternal, socioeconomic, and demographic data, it was possible to describe these Xavante children in relation to the presence of anemia (via hemoglobin levels). Anthropometric data were considered those used in human nutritional diagnosis, represented in this study by the P/I and BMI variables. Information related to the evaluation of individuals' health status was classified as clinical data, represented in this study by the variables hospitalization frequency, presence of overweight, presence of anemia, and serum hemoglobin level. Maternal data included all information related to the mothers of the children included in this study. Socioeconomic data encompassed information regarding the economic and social status of the child or group, represented in this study by the variables income, number of people per household, and village group location. Finally, demographic data referred to population characteristics such as age, sex, as well as the number of people per household and village location.

To develop an indicator capable of considering the historical process of territory occupation over time, as well as population growth and the emergence of new villages from a mother village, the ‘village group’ variable was created. This variable, defined in the study by Arantes et al. (2018), consists of three groups of villages determined according to a historical/temporal criterion of village foundation and is associated with a geographical location pattern within the IT.

The first subgroup (referred to as Group 1) consisted of two villages: one village, the oldest, which gave rise to all the others, and a second village that emerged in 2006 and remained very close to the first. Both are located in a less degraded Cerrado area and are more distant from regional urban centers. The second subgroup (referred to as Group 2) comprised two villages: one village that separated from the mother village in the early 1980s and a second village that emerged more recently from the former, established near the Rio das Mortes. Both are located in a lower, flatter region with poor drainage, characterized by “wet fields.” This area was previously occupied by farms whose reserves were incorporated into the territory during the IT demarcation process.

The third subgroup (referred to as Group 3) consisted of three villages: one older village, which originated from the mother village in the late 1980s, and two others that emerged from it through a fission process in the early 2000s. These villages are geographically close to each other and a few kilometers from the interstate highway and neighboring farms within the territory. They are also close to a village, a district of the municipality of Canarana, that is located on the highway margins.

### 2.4 Statistical Analyses

Descriptive analyses were conducted using relative and absolute frequencies to estimate the prevalence of anemia according to the selected variables. The chi‐square test was employed, except in cases where at least one category had an observation count of less than 5, in which case Fisher's exact test was used, with a *p* < 0.05 considered significant in both cases. To calculate the mean hemoglobin levels across the defined categories within each group of variables, the outcome was treated as continuous. In addition to the mean, the standard deviation was calculated, with a significance level of 5% (95% CI).

The remaining variables selected to describe the Xavante children were classified into three categories: child‐specific, maternal, and household‐related variables. The child‐specific variables were classified by age group, sex (female or male), the absence or presence of hospitalization within 12 months prior to data collection, and the absence or presence of a low WAZ. The maternal variables were categorized by age group, overweight status, as indicated by BMI, and anemia classification (WHO 2024). Finally, the household variables were categorized by village group, per capita income quartile, and the number of individuals living in the household.

#### 2.4.1 Path Analysis

**FIGURE 1:**
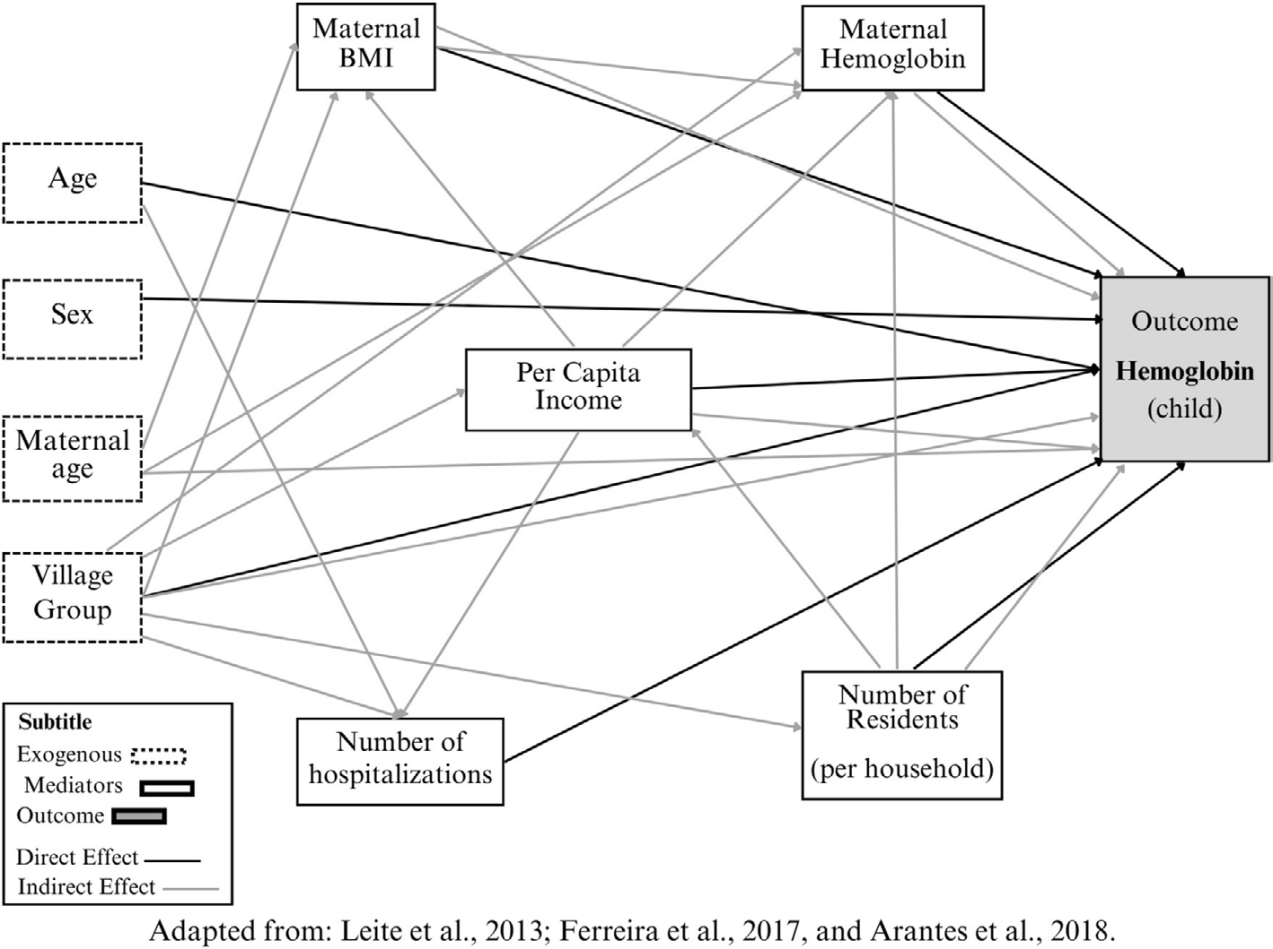
Hypothetical model to evaluate the association between the outcome and the socioeconomic, environmental, maternal, and individual characteristics of indigenous Xavante children between 6 months and 5 years, in relation to anemia status. Pimentel Barbosa Indigenous Land, Central Brazil, 2011. *Source:* Adapted from Leite et al. (2013), Ferreira et al. (2017), and Arantes et al. (2018).

In this stage of the study, the theoretical model was developed, adapted from Arantes et al. (2018), and based on the authors (Leite et al. 2013; Ferreira et al. 2017; Barreto et al. 2020), who worked with theoretical models on anemia and Indigenous child health in Brazil (Figure 1), to assess the relationships among the factors associated in determining anemia within the context of Xavante children. In this phase, the primary outcome considered was the child's hemoglobin level, which was treated as a continuous variable. All other variables in the model were also analyzed as continuous variables, except child sex and village group, which were treated as categorical variables, for which male sex and Group 1 were considered as reference categories. The exposure variables considered were age and sex, maternal age, and village group. The selection of variables was based on the researchers' prior experiences and studies that evaluated anemia among Indigenous populations in the country or health determinants in Xavante children (Arantes et al. 2018; Ferreira et al. 2012, 2017; Welch et al. 2009).

Figure 1 illustrates the direct and indirect relationships among observable variables, forming causal pathways within the model. The variables, represented by rectangles, form the trajectory of hypothetical associations with their arrows, starting from the set of exposures to the outcome. Direct and indirect effects were estimated via the robust maximum likelihood estimator (MLR). Standardized coefficients (*β*) were determined, and missing values were handled by full information maximum likelihood (FIML) without actual imputation. The adequacy of the necessary adjustments in this model was evaluated on the basis of five criteria: significance determined via the chi‐square test (*χ* ^2^/df ratio < 3.0), standardized root mean squared residual (SRMR ≤ 0.08), root mean squared error of approximation (RMSEA with 90% CI < 0.06), Tucker–Lewis index (TLI ≥ 0.95), and comparative fit index (CFI ≥ 0.95) (Hu and Bentler 1999). According to Hu and Bentler (1999), for a good model in practice, it is necessary for the model to meet at least two of the above fit indices.

### 2.5 Ethical Considerations

The study was approved by the Research Ethics Committee at the National School of Public Health (Escola Nacional de Saúde Pública), Oswaldo Cruz Foundation (Fundação Oswaldo Cruz), and the National Research Ethics Council (Conselho Nacional de Ética em Pesquisa) CONEP, process no. 25000.202987/2010‐14. Entry into the Indigenous reserve was authorized by community leaders in accordance with local protocols. The project was presented to residents and leaders of Indigenous reserve villages during community meetings. The village leaders signed a collective prior informed consent form for the residents in the event of approval. The participants were informed that they could refuse to participate in the research at any stage of the fieldwork. During home visits, any additional questions about the study were answered before data collection took place.

## 3 Results

At the time of data collection, the total population residing in the participating villages was 1337 individuals, of whom 311 (23.2%) were children under 5 years of age. Among these children, 281 (90.3%) were investigated, with ages ranging from 6 months to 5 years. Losses occurred due to data inconsistencies, typographical errors, or absence at the time of data collection. There were no refusals to participate.

Overall, 61.1% of the children had anemia. Among these children, 80.5% had mild anemia, and 33.7% had moderate or severe anemia. In children under 2 years of age, this percentage reached 78.1% (data not shown in the tables). Table 1 highlights the prevalence of anemia according to the children's individual and biological factors, as well as maternal and sociodemographic factors with respect to the household. The incidence of anemia decreased with increasing age and was significantly greater among children between 6 months and 1 year (78.1%) and those between 1 and 2 years of age (72.1%) (Table 1).

**TABLE 1:** Prevalence of anemia and hemoglobin levels among Xavante indigenous children between 6 months and 5 years according to individual, maternal, and household characteristics from the Pimentel Barbosa Indigenous Land, Central Brazil, 2011.

Variables	Total (100%)	No anemia, *n* (%)	Anemia, *n* (%)	*p*	Hemoglobin (g/dL)	
					Mean	95% CI
*Child*						
Age (months)						
≥ 6 to < 12	32	7 (21.8)	25 (78.1)	0.002	9.1	8.6–9.1
≥ 12 to < 24	61	17 (27.8)	44 (72.1)		9.6	9.3–10.0
≥ 24 to < 36	63	22 (34.9)	41 (65.08)		10.2	9.8–10.5
≥ 36 to < 48	43	22 (51.1)	21 (48.8)		10.7	10.4–11.1
≥ 48	53	30 (56.6)	23 (43.0)		11.1	10.8–11.4
Sex						
Female	129	49 (37.9)	80 (62.0)	0.763	10.2	10.0–10.5
Male	123	49 (39.8)	74 (60.1)		10.2	9.9–10.4
Hospitalization						
No	139	62 (44.6)	77 (55.4)	0.039	10.4	10.2–10.6
Yes	113	36 (31.8)	77 (68.1)		9.9	9.7–10.2
W/A						
≥ −2 *z* score	233	88 (37.7)	145 (62.2)	0.201	10.7	10.1–11.3
< −2 *z* score	19	10 (52.6)	9 (47.3)		10.2	10.0–10.3
*Mothers*						
Age (years)						
< 18	42	16 (38.1)	26 (61.9)	0.967	10.2	9.7–10.6
≥ 18 to < 30	126	50 (39.6)	76 (60.3)		10.2	10.0–10.5
≥ e30	84	32 (38.1)	52 (61.9)		10.2	9.93–10.5
BMI (kg/m^2^)						
Eutrophy (≥ 18.5 to < 25)	79	33 (41.7)	46 (58.2)	0.526	10.1	9.8–10.5
Overweight (≥ 25)	173	65 (37.5)	108 (62.4)		10.2	10.0–10.4
Anemia (Hb g/dL)						
Absent (≥ 12)	85	35 (41.1)	50 (58.8)	0.828	10.5	10.2–10.8
Mild (≥ 11 to < 12)	56	19 (33.9)	37 (66.0)		10	9.6–10.4
Moderate (≥ 8 to < 11)	102	40 (39.2)	62 (60.7)		10.1	9.92–10.4
Severe (< 8)	9	4 (44.4)	59 (55.5)		9.8	8.79–10.8
*Residence*						
Village group						
1	134	70 (52.2)	64 (47.7)	< 0.001	10.7	10.5–10.9
2	46	14 (30.4)	32 (69.5)		10.1	9.7–10.5
3	72	14 (19.4)	58 (80.5)		9.4	9.0–9.7
Per capita income (quartiles)						
1°	59	23 (38.9)	36 (61.0)	0.862	10.1	9.8–10.5
2°	60	26 (43.3)	34 (56.6)		10.1	9.7–10.5
3°	67	25 (37.3)	42 (62.6)		10.2	9.9–10.6
4°	66	24 (36.3)	42 (63.6)		10.3	10.0–10.6

*Note:* Chi‐square and Fisher's exact tests (*p* < 0.05); legend of village groups: Group 1 (Pimentel Barbosa and Etênhiritipá), Group 2 (Caçula and Wedezé), and Group 3 (Tanguro, Asereré and Reata).

Children who had been hospitalized at least once in the year prior to the study had a higher prevalence of anemia (68.1%). In Group 3, this prevalence reached 80.5%, whereas Group 1, which had the highest number of evaluated children, had the lowest prevalence (47.7%). These differences were significant (*p* < 0.001) (Table 1).

According to Table 1, the majority of Xavante households in the TI Pimentel Barbosa area that housed children up to 5 years old had between 10 and 20 residents (*n* = 139). Compared with other categories, the lowest prevalence of anemia (57.3%) was found among children from households with fewer than 10 people. The highest prevalence of anemia was observed among children from more populous households (69.2%; with more than 20 residents) (Table 1).

**FIGURE 2:**
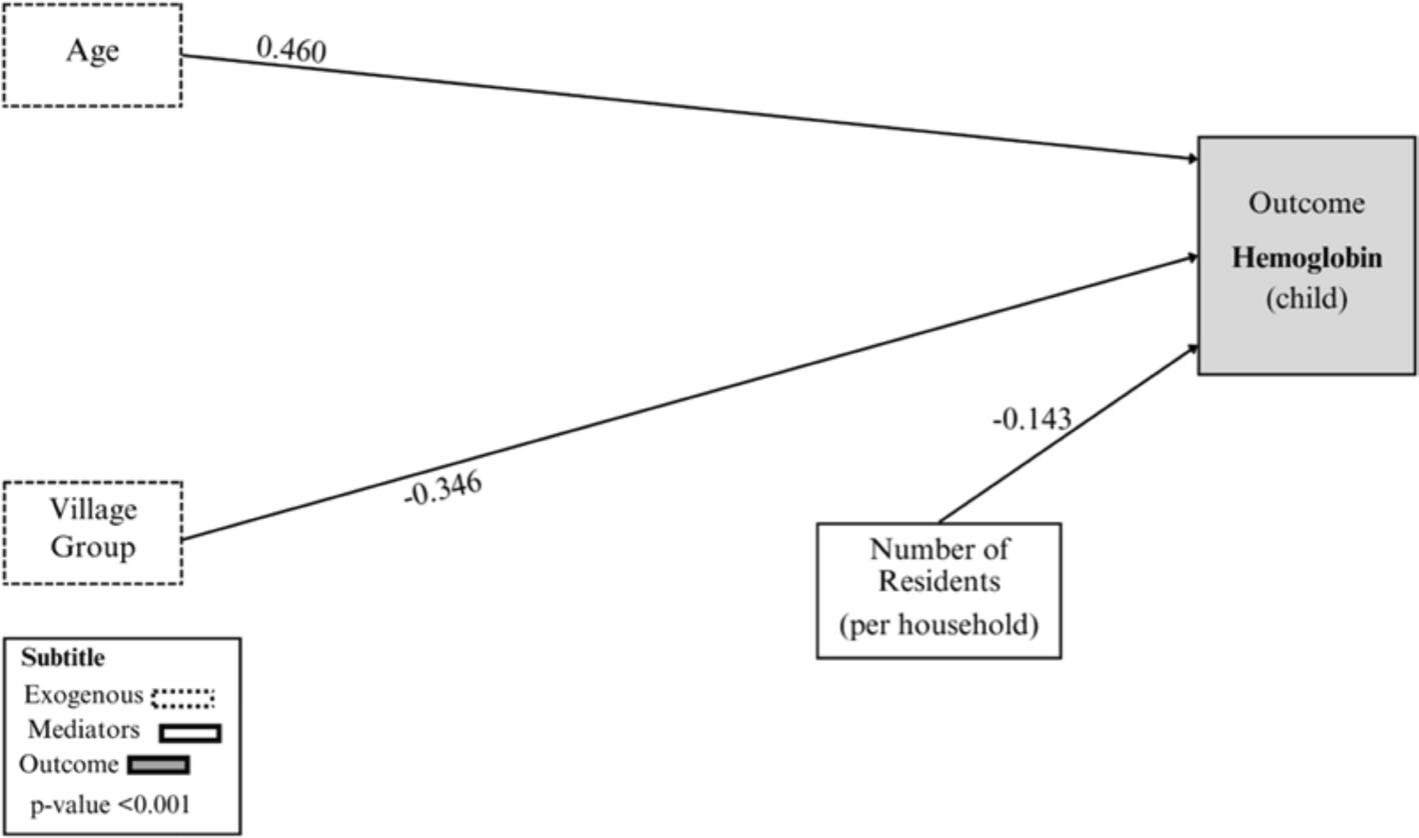
Diagram of the final model of direct associations between the outcome and the socioeconomic, environmental, maternal, and individual characteristics of Xavante indigenous children between 6 months and 5 years, in relation to anemia status. Pimentel Barbosa Indigenous Land, Central Brazil, 2011. *Source:* Author's own work.

Path analysis revealed that the final model (*χ* ^2^/df ratio = 0.3517, SRMR = 0.032, RMSEA (95% CI) = 0.056, TLI = 0.978, and CFI = 0.990) had an acceptable fit (data not shown in the tables/figures). Figure 2 illustrates the model and its direct associations with the outcome, along with the standardized coefficients (*β*) for the significant relationships. Child age was directly related to the outcome. As age increased, the hemoglobin level also increased (*β* = 0.460). With respect to the number of household members, as the number of individuals increased, the hemoglobin levels decreased (*β* = −0.143).

Figure 2 also shows the links and directions of relationships between the outcome and the village groups. Considering that the village groups (categorized as Groups 1– 3), formed a sequence from the oldest and most developed group (Group 1—reference category), to the newest and least developed group, the newer the village were, the lower the children's hemoglobin levels (*β* = −0.346) in relation to the reference group. This may occur because the villages in Group 1 (Pimentel Barbosa and Etênhiritipá villages) have local schools, health posts, and their own agricultural lands, which have been established over the years. These are considered “mother villages,” as they gave rise to the more recent villages that have not yet fully developed and, therefore, have limited access to these essential resources.

Other significant associations between variables, though not necessarily with the outcome, that were identified by the model, as well as their standardized coefficients, are detailed in Table 2. Notably, various relationships between village groups and socioeconomic conditions, maternal health, and child health are highlighted.

**TABLE 2:** Final adjusted model: Coefficient (*β*), standard error (SE), and *p* value for the association between hemoglobin and individual, maternal, and household characteristics of Xavante Indigenous children, between 6 months and 5 years, from the Pimentel Barbosa Indigenous Land, Central Brazil, 2011.

Associations	*β*	SE	*p*
Hemoglobin—age	0.460	0.047	< 0.001
Hemoglobin—no. of residents	−0.143	0.046	0.002
Hemoglobin—village group	−0.346	0.047	< 0.001
Per capita income—village group	−0.126	0.053	0.018
Hospitalization—village group	0.211	0,056	< 0.001
Hospitalization—age	−0.105	0.055	0.055
Maternal BMI—maternal age	0.271	0.058	< 0.001
Maternal BMI—village group	−0.127	0.055	0.021
Maternal hemoglobin—per capita income	0.145	0.046	0.002
Maternal hemoglobin—village group	−0.127	0.057	0.026

*Note: p* < 0.01.

## 4 Discussion

These findings suggest that anemia is not only prevalent among Xavante children but also associated with socioeconomic, demographic, and historical determinants. The higher prevalence of anemia found in children under 2 years of age is consistent with findings from various studies in Brazil and Latin America for this age group (Ferreira et al. 2024, 2017; Rosas‐Jiménez et al. 2022). Prevalence rates of 60%–70% among Indigenous children under 2 years of age in Latin America are not uncommon (De Louize et al. 2022; Ferreira et al. 2024). The anemia prevalence reported in this study (61.1%) is higher than that reported among Indigenous children in the Central‐West Region of Brazil (51.5%) (Leite et al. 2013). Moreover, the prevalence of anemia in this study is approximately 10 times greater than the prevalence of anemia in non‐Indigenous Brazilian children (10%) (UFRJ 2021). Importantly, population‐based nutritional surveys in Brazil do not include the Indigenous group in a representative manner, and recent research on anemia among Indigenous peoples is rare.

This underscores the importance of studies aimed at understanding this dynamic specifically within Indigenous populations, such as that in this study, which is underscored. Hemoglobin levels in Xavante children increase with age. Therefore, younger age is associated with a lower hemoglobin level, which may reflect greater vulnerability, as well as the conditions of prenatal care, birth weight, breastfeeding status, a higher incidence of infectious and parasitic diseases, and unmet micronutrient needs (Fávaro et al. 2019; Ferreira et al. 2024, 2021; Souza et al. 2021).

Moreover, it is known that during the first 2 years of life, the demand for iron increases due to rapid growth and child development, regardless of race. Consequently, serum hemoglobin levels may decrease, and the risk of anemia may rise (Ferreira et al. 2024; WHO 2024). Therefore, the presence of anemia in Indigenous children under 2 years old becomes even more concerning when considering their already high rates of infant mortality, malnutrition, and food insecurity. Furthermore, they live in a context of uncertainty regarding access to food, healthcare, adequate sanitation, and the enforcement of public policies. As a result, anemia levels among Indigenous children in the country tend to increase.

Understanding the relationships among the determinants of anemia in this context remains a challenge, regardless of the ethnic group, as anemia involves complex, multifactorial, and multicausal nutritional issues (Balarajan et al. 2011; WHO 2017). Considering this complexity within Brazilian Indigenous populations is even more challenging due to the diverse sociocultural, historical, and environmental contexts, which can directly influence a child's nutritional status. The literature includes some comparative studies between Indigenous and non‐Indigenous populations exploring this relationship in an attempt to better understand possible causal or relational factors (Pereira et al. 2020), although often with simpler techniques.

In this context, path analysis, the methodology used in the study, is an extension of multiple regression, allowing analysis of models with more complex and multicausal outcomes, such as anemia. Thus, we were able to evaluate unique indicators for each of the variables in the model, enabling more precise estimates of the effects between the dependent and independent variables.

As an example of the applicability of this technique, we found that most Xavante mothers were overweight (*n* = 173), and 62.4% of these mothers had children with anemia. The association between overweight and anemia among mothers and their children corroborates findings in studies of both Indigenous and non‐Indigenous peoples. The food environment in this context suggests the presence of a double burden of malnutrition, both at the individual level among these mothers and possibly at the household level (Fávaro et al. 2019; Popkin et al. 2020; Gedfie et al. 2022). Additionally, another aspect reflecting the influence of household conditions on Xavante children's health was the association between hemoglobin levels and the number of individuals living in the household. Having greater hemoglobin levels was associated with being from a less populous household. This finding supports the high prevalence of anemia (69.2%) observed in Xavante households with more than 20 residents.

It is well known that unfavorable socioeconomic and demographic factors, whether in Indigenous or non‐Indigenous health contexts, can influence children's nutritional status, especially those under 5 years of age (Ferreira et al. 2017; Ghosh 2023; Oliveira et al. 2022). However, these factors are compounded by historical conditions of discrimination, ethnic‐racial inequalities, and health inequities, which exacerbate nutritional conditions not only for children but also for the entire population (Ferreira et al. 2024; Pedraza et al. 2014; Santos et al. 2018).

To understand some of the dynamics involved in these overlapping inequalities, the prevalence of childhood anemia was assessed according to village groups. Group 1, which had the lowest prevalence of anemia, consisted of the oldest and most established villages that gave rise to the other villages. Villages in Group 3, which had the highest prevalence of anemia, were located closer to the interstate highway, a small town, and operational farms.

Consequently, the association between hemoglobin levels and village groups, as revealed by path analysis, revealed that Group 1 was associated with higher hemoglobin levels. Groups 2 and 3, with increasing distance from the historical‐social origin group, were associated with lower hemoglobin levels. Factors such as the age of the village, internal and organizational divisions, the social structures of the villages within each group, and the degree of proximity to urban centers may be related to this association (Arantes et al. 2018).

The distance of villages from urban centers can lead to changes in lifestyle, partly explaining the dynamics between residential location and the prevalence of childhood anemia. However, newer villages with lower population densities tend to have even less access to healthcare services, experience practical subsistence challenges, and have potentially poorer dietary practices (Garnelo and Pontes 2012; Santos et al. 2018). Additionally, these villages may have a worse infrastructure for ensuring food and nutritional security and health, such as health posts and Indigenous schools. Communities such as Pimentel Barbosa and Etenhiritipá, and others founded earlier, have had a health post and schools that serve Indigenous children in the communities for a longer time. In addition, the communities have different road distances from commercial centers, which can influence access to food (Welch and Coimbra Jr. 2022).

As a result, newer village groups had a greater frequency of hospitalizations among children under 5 years of age. Recurrent hospitalizations, infections, and other illnesses are strongly associated with increased anemia incidence (Escobar et al. 2015; Caldas et al. 2023). Although hospitalization frequency was not directly related to anemia in this study, it was indirectly related to the village group and, consequently, to hemoglobin levels. In this context, the study by Leitão et al. (2011) highlighted that children who had at least one hospitalization episode in their lifetime were at greater risk of anemia than children who had never been hospitalized (Leitão et al. 2011).

The ineffectiveness and inefficacy of certain public health policies targeting Indigenous populations in Brazil are noteworthy, mirroring challenges faced by other South American Indigenous groups (Khambalia et al. 2011; Goetz and Valeggia 2017; Santos et al. 2022). Despite the existence of a national iron supplementation policy theoretically inclusive of Indigenous communities, several factors hinder the continuity and monitoring of anemia prevention efforts beyond biological considerations. While iron supplementation is essential, it alone cannot resolve the issue in contexts such as that of the Xavante, where health inequities, inadequate sanitation, and food insecurity are prevalent.

It is worth noting that the cross‐sectional design does not allow for causal relationships, and the limited number of covariates, including the absence of information on prenatal care, iron supplementation during pregnancy, breastfeeding practices, complementary feeding, and levels of other micronutrients, restrict some possible interpretations of the analysis, especially in the path analysis approach.

Another limitation of this study was the inability to perform structural equation modeling due to variable parameter constraints. However, path analysis in cross‐sectional studies can be highly effective when combined with theoretical grounding and the previous experiences of the researchers involved. Importantly, no studies have explored the relationship between anemia in Xavante children through a more robust statistical analysis that is capable of considering the entirety of an Indigenous reserve and its sociopolitical and organizational divisions.

## 5 Conclusion

The high prevalence of anemia identified among Xavante children under 5 years of age has determinants that function as biological, socioeconomic, and demographic agents, influencing the outcome. Thus, strategies to reduce the prevalence of anemia in the context of Indigenous health should encompass not only nutrition and health spheres but also aspects related to the environmental and social context.

Research on anemia in Indigenous populations of the Amazon region needs to be expanded, with systematic consideration of local and global socioeconomic and historical factors that influence the human biology of these populations. Additionally, broadening the traditional focus of anemia research is essential to developing analytical frameworks that better capture the processes of change among Indigenous peoples. Anemia among the Xavante and other Indigenous peoples in Brazil should be monitored, treated, and evaluated within the context of the numerous historically unfavorable conditions, including situations of ethnic‐racial nonrecognition, a lack of access to basic human rights, and difficulties in securing territorial and sanitary rights. Public policies targeting this population should not be prioritized only during pandemics or humanitarian emergencies. To reduce health inequities for these people, the numerous processes of colonization that have caused disruptions in traditional lifestyles, the loss of territories, environmental degradation and invasion, and persistent ethnic‐racial discrimination must be addressed.

Another important factor may relate to community‐level dynamics and household size, both of which can influence the anemia profile in this group, underscoring the significance of the social context in understanding the disease's dynamics. While large households may pose challenges to food and nutrition security, they also serve as crucial social support networks for Indigenous communities. Therefore, examining community and household structures is essential for future research on anemia and human biology among South American Indigenous populations.

Despite advancements in access to healthcare driven by public policies in the first decade of the 21st century, as well as the growing presence of Indigenous leadership in the health sector, most Indigenous communities continue to experience deficits in healthcare and education services, various forms of food insecurity, high mortality rates, and instability in programs and policies.

## Author Contributions

A.A.F., J.R.W., C.E.A.C., and R.A. participated in the conception of the study, collection of data in the field, and drafting of the manuscript. A.A.F. also contributed to data analysis. L.L.A.P. and J.B.L. contributed to the study design, analysis, and interpretation of the data, and writing of the paper. All authors participated in the revision of the manuscript and approved the version submitted for publication.

## Conflicts of Interest

The authors declare no conflicts of interest.

## Data Availability

The data that support the findings of this study are available from the corresponding author upon reasonable request.
